# Multidisciplinary discovery of ancient restoration using a rare mud carapace on a mummified individual from late New Kingdom Egypt

**DOI:** 10.1371/journal.pone.0245247

**Published:** 2021-02-03

**Authors:** Karin Sowada, Ronika K. Power, Geraldine Jacobsen, Timothy Murphy, Alice McClymont, Fiona Bertuch, Andrew Jenkinson, Jacinta Carruthers, John Magnussen

**Affiliations:** 1 Department of History and Archaeology, Macquarie University, Sydney, New South Wales, Australia; 2 McDonald Institute for Archaeological Research, Department of Archaeology, University of Cambridge, Cambridge, United Kingdom; 3 Centre for Accelerator Science, Australian Nuclear Science and Technology Organisation, Sydney, New South Wales, Australia; 4 Department of Earth and Environmental Sciences, Macquarie University, Sydney, New South Wales, Australia; 5 Faculty of Medicine and Health Sciences, Macquarie University, Sydney, New South Wales, Australia; Hebrew University, ISRAEL

## Abstract

CT scans of an unnamed mummified adult from Egypt, now in the Chau Chak Wing Museum, University of Sydney (NMR.27.3), reveal it to be fully sheathed in a mud shell or carapace, exposing a mortuary treatment not previously documented in the Egyptian archaeological record. The carapace was placed between layers of linen wrappings thus it was not externally visible. Radiocarbon dating of textile samples provide a range of c.1370–1113 cal BC (95.4% probability), with a median date of 1207 cal BC. When assessed against mummification techniques of the era, the individual is placed in the late 19th–20th Dynasty, at the later end of this date range. Multi-proxy analysis including μ-XRF and Raman spectroscopy of carapace fragments from the head area revealed it to consist of three layers, comprising a thin base layer of mud, coated with a white calcite-based pigment and a red-painted surface of mixed composition. Whether the whole surface of the carapace was painted red is unknown. The carapace was a form of ancient conservation applied subsequent to post-mortem damage to the body, intended to reconfigure the body and enable continued existence of the deceased in the afterlife. The carapace can also be interpreted as a form of elite emulation imitating resin shells found within the wrappings of royal bodies from this period.

## Introduction

Hardened ‘shells’ within the linen wrappings of mummified people from ancient Egypt are known from the study of elite mummification of the late New Kingdom to the 21st Dynasty (c. 1294–945 BC). In the first systematic examination of the mummified bodies of royal individuals housed in the Egyptian Museum, Cairo, layers of ‘fine linen and resinous paste’ were reported [1 (p 99, 103, 105), 2 (p 61)]. Yet despite the plethora of scientific studies since undertaken on other bodies, such features are rarely discussed in the literature.

Radiological investigations on the mummified body of an adult individual in the Nicholson Collection of the Chau Chak Wing Museum, NMR.27.3, resulted in the discovery of a rare painted carapace within the wrappings. Additional scientific techniques exposed complex post-mortem treatment and further confirmed that the body was not the named individual on the coffin. When set against established afterlife beliefs and developments in elite mummification techniques, the carapace can be understood as both a practical and symbolic funerary artefact.

**Fig 1:**
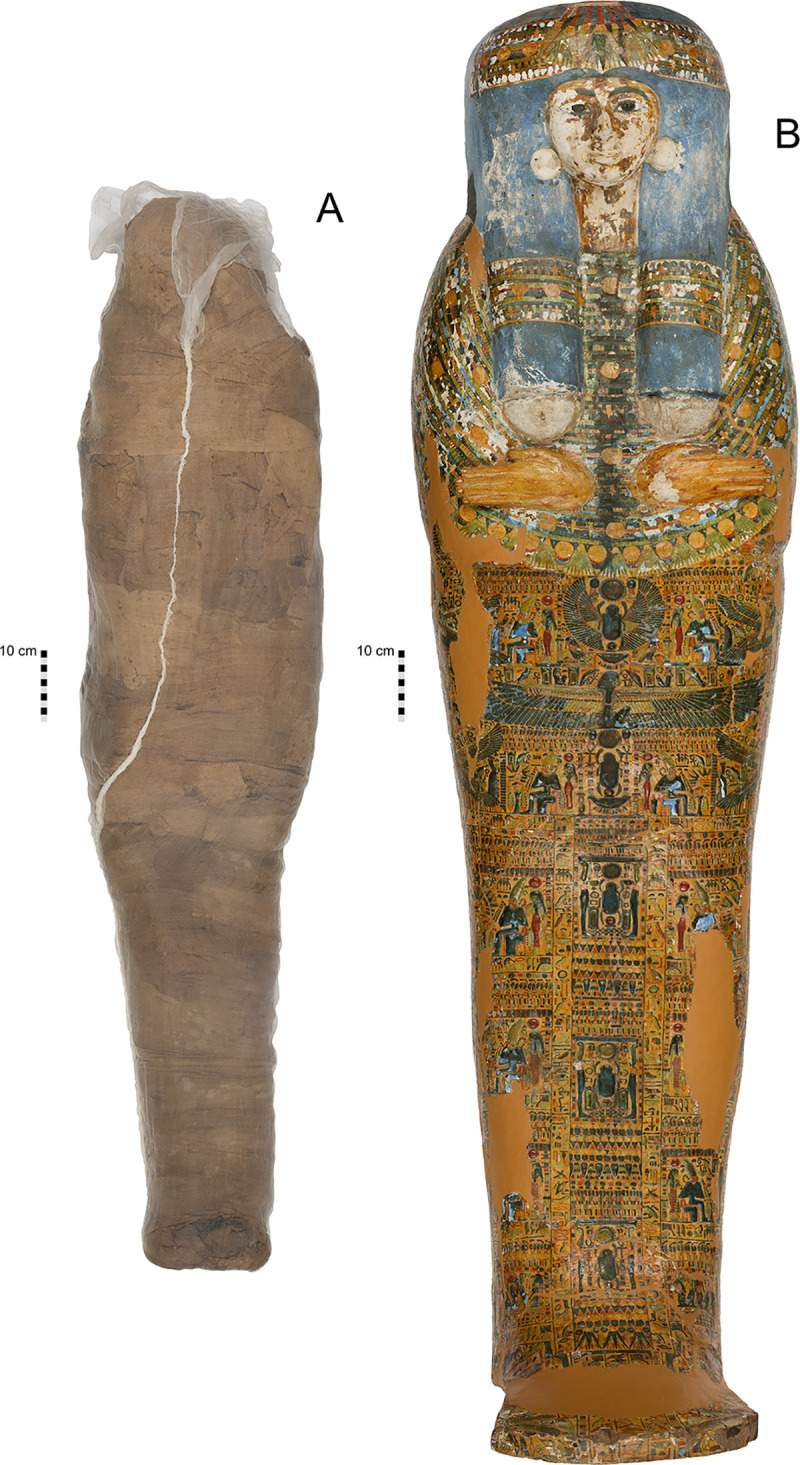
Mummified individual and coffin in the Nicholson Collection of the Chau Chak Wing Museum, University of Sydney. A. Mummified individual, encased in a modern sleeve for conservation, NMR.27.3. B. Coffin lid, NMR.27.1. (Published under a CC BY license, with permission from the Chau Chak Wing Museum, original copyright 2019).

The mummified body was acquired by Sir Charles Nicholson during his trip to Egypt in 1856–1857 [3]. Little is known about its acquisition, as is sadly the case for many human bodies procured in Egypt by European and American collectors in the 19th and early 20th centuries. The mummified body, the lidded coffin in which it rested (NMR.27.1) and its mummy board (NMR.27.2) originated in Western Thebes and was likely purchased in Luxor. The ensemble was donated to the University of Sydney by Nicholson in 1860 (Fig 1).

The coffin inscription identifies the owner as a titled woman named Meruah (or Meru(t)ah [4 (p 170)]). Niwiński dated the coffin to the mid–late 21st Dynasty, c. 1010–945 BC [4 (p 170), 5 (p 228–233)]. New research places it around c. 1000 BC based on the iconography of its decoration [6 (p 171–176)].

The adult body in the coffin was assumed to be the female named on its exterior. Although DNA results identified the body as a male [7], these findings are challenged by the current study. Further evidence reveals that the date of the individual is earlier than that of the coffin. Local dealers likely placed an unrelated mummified body in the coffin to sell a more complete ‘set’, a well-known practice in the local antiquities trade [8].

## Methods and results

### Radiological analyses

#### Radiological methods

The shell was discovered in 1999 during a computed tomography (CT) scanning project led by the first author [7]. On 21 December 1999, Professor Allan Spigelman (Faculty of Medicine, University of New South Wales, Sydney) extracted four pieces of the shell under sterile conditions through a small incision made into the wrappings from the left inferolateral aspect of the face. On the CT workstation, these fragments were originally regarded as bone, appearing as loose pieces around the head.

**Fig 2:**
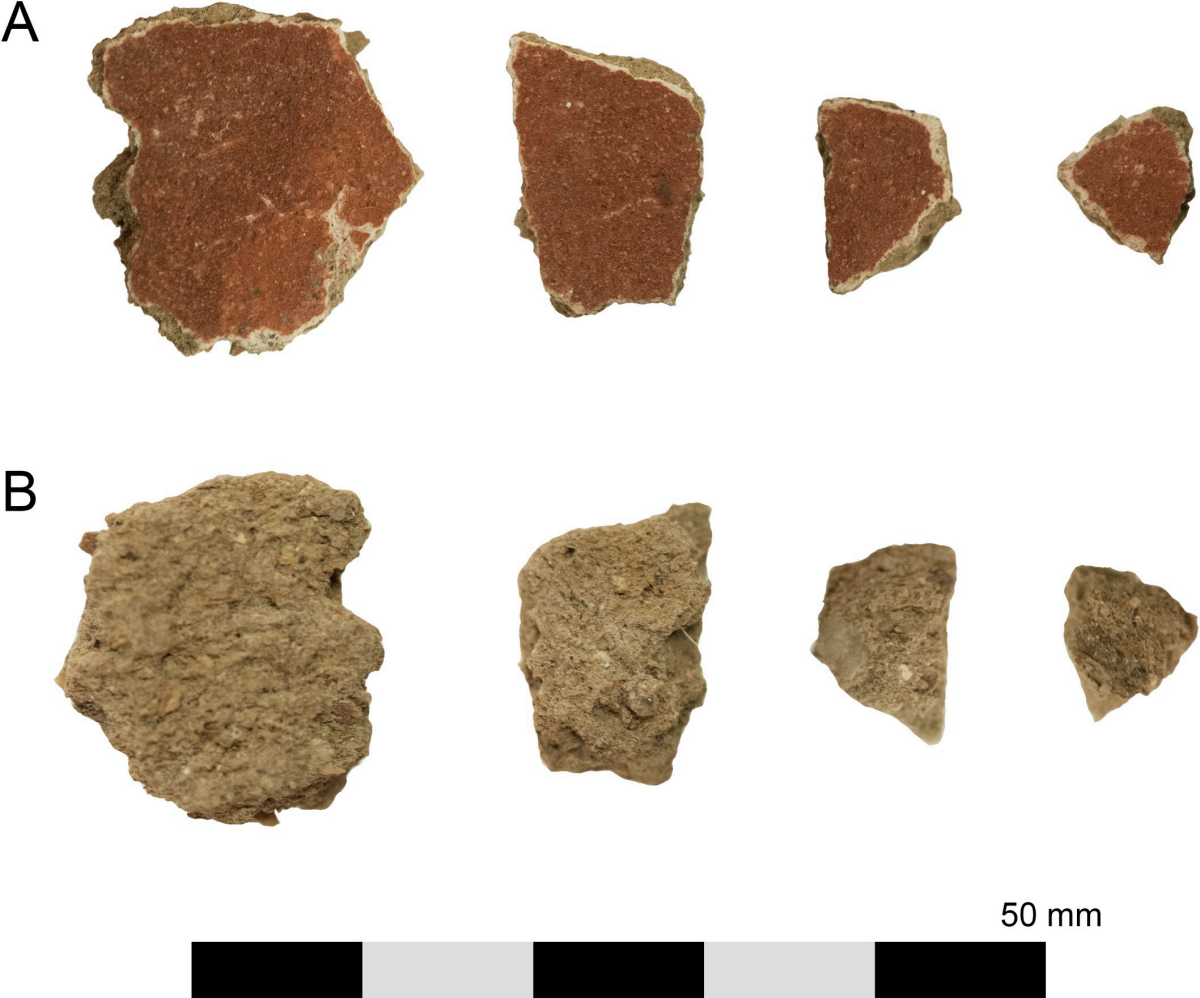
Carapace fragments NMR.27.13a–d from left to right (photographs by K. Sowada). A. Recto, showing red pigment. B. Verso, showing mud base.

**Fig 3:**
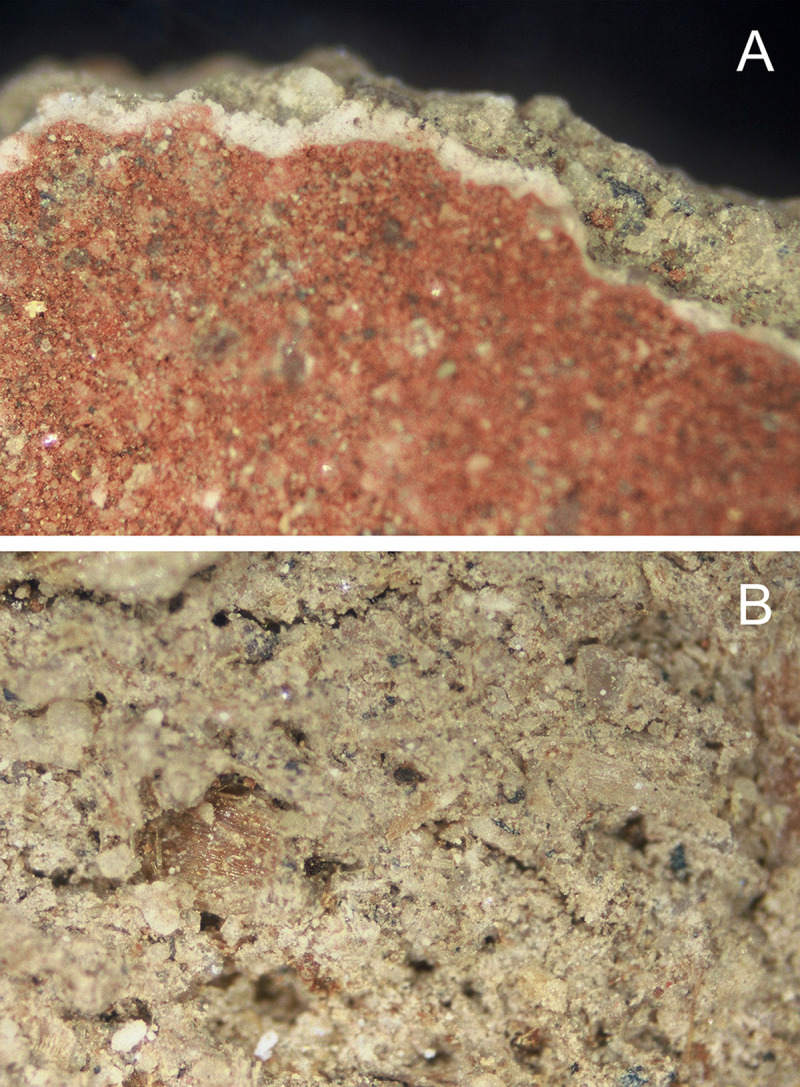
Carapace fragment NMR.27.13a. Magnification 7.8x (micrographs by R. Oldfield). A. Recto. Mud base, coated with white pigment base coat and red pigment. B. Verso. Note straw fragment in matrix.

The largest fragment measured 10.0 mm x 9.0 mm. The pieces are identical in manufacture, consisting of an uneven layer of unfired fine brown clay or mud containing sub-rounded sand, fine black-grey stone and fibrous material no more than 2.5–3.5 mm thick, coated on one side with a thin layer of white plaster or pigment, which was further coated in red pigment (Munsell 2.5YR 5/6 [9]) (Figs 2 and 3). Fine organic matter, possibly straw or chaff, is visible in the matrix. The verso of one fragment bore a faint textile impression, indicating that the substance was applied to the wrappings while the clay was still damp and pliable.

In December 2017, the body was re-scanned, providing significantly improved images. Although ethics approval was obtained for the original study in 1998 (University of Sydney Human Ethics Committee Ref. 98/3/31), no permits were required for the described study, which complied with all relevant regulations. CT scans were performed on a GE Lightspeed Revolution CT (General Electric, Milwaukee, USA) and reviewed on a GE Advantage Workstation. Helical acquisitions were performed at 120 kVp with automated dose modulation (average 230 mAs) with reconstructions performed at 0.625 mm spacing using Standard, Bone and Bone Plus kernels, giving 512 x 512 matrices of 0.9 x 0.9 mm per pixel for full field of view reconstructions and 0.23mm for small field of view reconstructions in areas of interest.

In the 18 years since the prior CT examination there has been significant technological innovation which has led to the dramatic improvement in image quality, spatial resolution and contrast resolution. The original scans performed in 1998 and 1999 consisted of 10mm axial slices through the entire body (1998), each slice taking 1.8s, and with improved technology in 1999, 3mm slices were acquired through the head and neck, with further imaging limited by scan time and limited x-ray tube output. Such thick slices were not suitable for multiplanar reformatting or for 3D / volumetric reconstructions.

When the bodies were re-scanned in 2017, the GE Lightspeed Revolution CT could acquire 256 slices (16cm) in one rotation, covering up to 437mm/sec with 0.23mm spatial resolution. With the use of advanced iterative reconstruction techniques, this provides for greatly improved soft tissue or low-contrast resolution [10]. Differentiating fine structures requires high spatial resolution and distinguishing adjacent structures of similar density requires improved low-contrast resolution. Both of these factors come into play when considering the ability to distinguish individual layers of linen wrapping (spatial resolution) and differentiating the density of the wrapping from that of a mud or resin carapace (low-contrast resolution). It is these two types of resolution which have improved the most since the introduction of the early multislice scanners in 1999 and which have contributed to the ability of this study to far better characterise discrete layers in the wrapping around the body.

To ensure optimal image acquisition during this study, a moderately low energy of 120 kVp was chosen and a relatively high dose scan technique used. The increased dose, while of no concern to an inanimate object and far below levels that may cause structural damage, gives improved image noise characteristics and thus greatly enhances the ability to confidently distinguish adjacent structures of otherwise similar density, such as the carapace from the underlying desiccated tissues.

The use of the GE Advantage Workstation provided advanced 3D analysis and visualisation capabilities, not possible in 1999 due to limitations in computing power and also the comparatively thick slices of data generated. 3D workstations such as this allow the operator to define density boundaries, generating reconstructions of objects based upon the virtual surfaces that they create and viewing those objects from any angle or direction. Furthermore, virtual dissections can be performed, with overlying or overlapping structures relatively easily removed to reveal internal features within objects, ideal for complex entities such as this mummified individual.

#### Anatomical position

**Fig 4:**
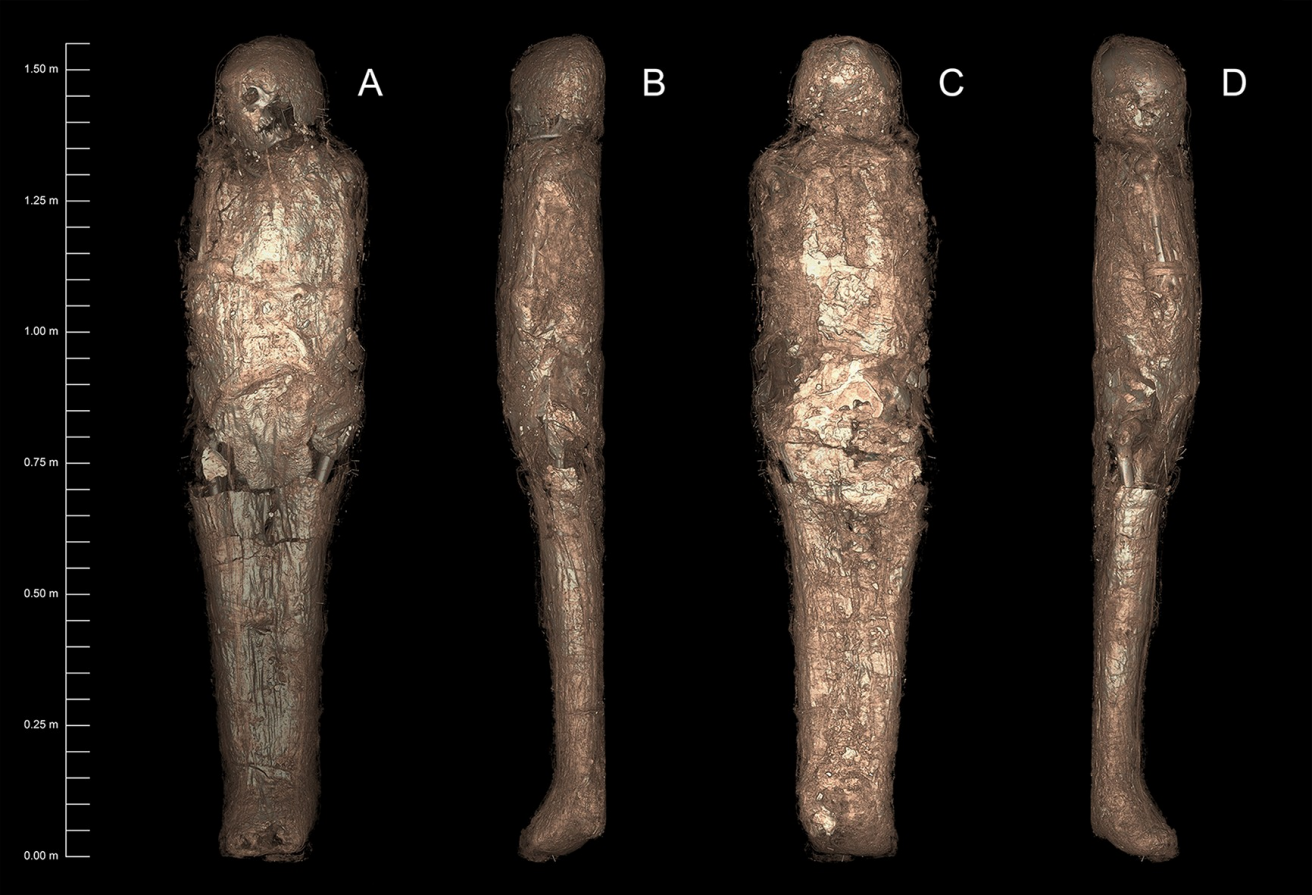
3D-rendered CT images of mummified individual (NMR.27.3) showing the carapace and broken sections (stitched image). A. Anterior. B. Lateral left-hand side. C. Posterior. D. Lateral right-hand side. (courtesy Chau Chak Wing Museum and Macquarie Medical Imaging).

**Fig 5:**
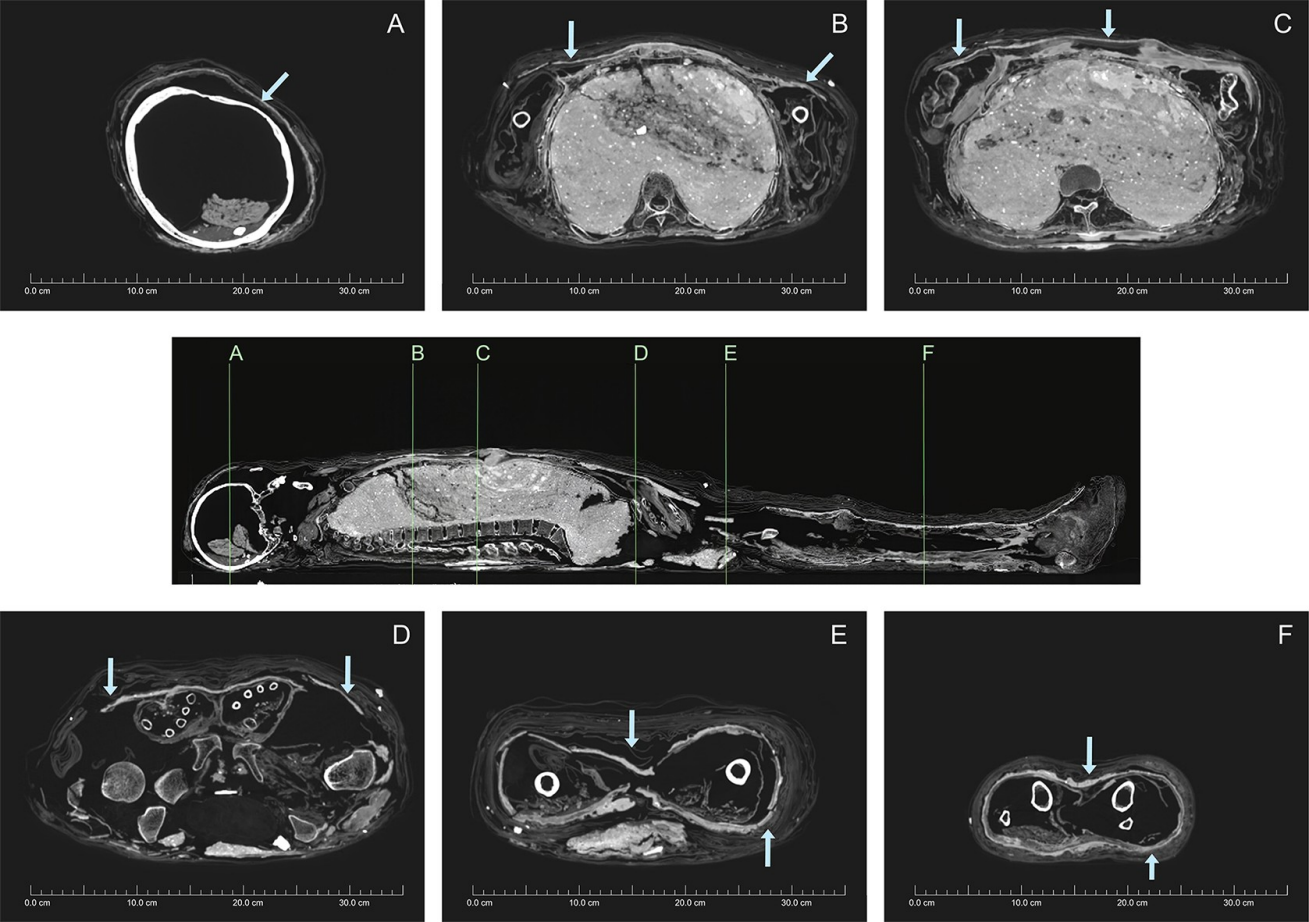
Axial CT images of mummified individual (NMR.27.3) showing transverse views of the carapace at different locations. The carapace is a thin white line under the wrappings (arrowed) (courtesy Chau Chak Wing Museum and Macquarie Medical Imaging).

The individual is observed to be in a supine position (Figs 4 and 5). The cranium and mandible are both present; however, they are not in articulation, nor is the cranial base in articulation with the first cervical vertebra. The cranium is rotated approximately 60° to the right and the mandible is rotated approximately 20° to the left. The vertebral column is disarticulated above C6. The upper cervical spine is disarticulated from the base of the cranium and lies in a transverse and posterior position inferior to the cranium. The upper cervical spine is no longer articulated and the attested elements (C1, C2, C3) are disorganised and fragmented. A number of maxillary and mandibular anterior teeth have been exfoliated post-mortem and moved to the posterior aspect of the body at this level.

At the base of the neck (at the level of C6) the wrapping presents as loosely folded outer layers superficial to the carapace. Tightly uniform wrapping is observed at the level of the chest. The upper arms are directly alongside the torso; the left and right forearms are slightly flexed at the elbows, orientated towards the midline. The left and right hands meet below the pubic symphysis, with the right hand placed underneath the left, between the upper thighs. Both hands are complete and intact.

The left and right legs are extended, and the feet are slightly hyperextended. Although the right femoral head is disarticulated, the right thigh remains in anatomical position. The distal right femur shows signs of post-mortem damage, as the supracondylar aspect and patella are both absent. The left thigh is internally rotated, with disarticulation observed at the level of the left knee. The left lower leg is disarticulated proximally, and the fibula has moved posteriorly, although it returns to anatomical position at the level of the ankle. Discontinuities are also observed in the dermal layers of the left calf. The left tibiofibular syndesmosis is disrupted, and the left subtalar and mid-tarsal joints are disarticulated. The left patella is observed at the level of the left ankle. The right patella is also disarticulated and is observed inferior to the right femoral head. The right tarsals, metatarsals and phalanges are all present but disarticulated and no longer in anatomical position. The right phalanges are grouped together but not in articulation with the metatarsals, except in the case of the great toe (*hallux*) which remains in articulation on both sides.

Fractures are observed through the greater trochanter of the right femur and lateral epicondyle of the right humerus. These fractures are designated as post-mortem as they exhibit no evidence of healing or periosteal reaction and are in direct association with disruptions in the overlying soft tissue and wrappings. The carapace is also disrupted in these regions and rolled textile has been placed around the damaged areas. Axial sections reveal that the thoracic cavity has been densely packed (Fig 5B and 5C). Two layers of packing are visible, representing materials of slightly different densities. The packing explains the heavy weight of the body, noted while moving it even before conducting any scans.

#### Demographic information

The CT scans were analysed to determine age and sex. The individual’s age was estimated to be a Young Middle Adult (26–35 years) via appraisal of cranial and post-cranial indicators, including the complete eruption of all permanent maxillary and mandibular dentition and closure of molar apices; complete fusion of the spheno-occipital synchrondrosis, bilateral medial clavicular epiphyses, proximal and distal epiphyses of all long bones of the upper and lower limbs, anterior aspects of extant first-to-third sacral segments, and bilateral iliac crest apophyses and acromial epiphyses [11, 12].

Biological sex was assessed via analyses of the osseous secondary sexual characteristics of the ossa coxae, cranium and mandible following standards established within Buikstra & Ubelaker 1994 [11] and Mitchell & Brickley 2017 [12]. The individual was determined to be a ‘probable female’. External genitalia were not observed on the scans, and internal reproductive organs were not retained. The radiological finding of this individual as a ‘probable female’ contrasts with the male molecular sex determination offered in previous research [7]. Considering the strength of expression of the secondary sexual characteristics, the present study proposes revision of the previous finding, and suggests that aDNA obtained from the sample may have been contaminated.

Detailed study of the skeletal remains and pathologies will be published in a separate paper.

#### Carapace description

Observational analysis of the CT images demonstrates that for the most part the carapace appears to have been applied in contiguous sheets, with some areas demonstrating a layering consistent with subsequent application of additional sheets of material. The carapace is observed to extend from the cranial vertex to below the distal pedal phalanges (Figs 4 and 5). The carapace is contiguous around the vertex, then interrupted around the right posterolateral aspect of the cranium for a short distance before another defect appears over the left malar eminence. It is then completely absent at the level of the mandible. The textile also presents a shear-plane at the level of the sixth cervical vertebra. The carapace is partially re-established at the level of the shoulders and base of the neck, becoming largely intact anteriorly at the level of the sternal notch. At the mid-sternal level, defects are observed posterolaterally and then laterally on the right side, becoming extensive throughout the expanse of the chest. This damage aligns with the aforementioned post-mortem skeletal damage on the same side. Furthermore, the right arm appears to have been rewrapped. The carapace is largely complete at the level of the iliac crest on the right side, but another defect is visible posterolaterally on the left. At the level of the femoral heads, a large defect is observed in the carapace, aligning with the post-mortem fracture of the right greater trochanter. Additional packing material of various densities is also observed in this region. The left side of the carapace in this region is collapsed and discontinuous but present. The carapace is contiguous from the proximal third of the thigh to the tibial plafond, becoming discontinuous posteriorly then anteriorly at this level before reforming at the toes. Apart from a defect anteriorly and distally in the midline, the carapace is continuous around the feet.

### Carapace scientific analyses

#### Dimensions

The carapace is variable in thickness (Table 1); some parts feature higher density components, suggesting possible inclusions within the groundmass, or intentional surface inclusions. Generally, the carapace is thicker posteriorly and inferiorly; it is very thin over the head and upper torso, and thicker over the thighs and more inferiorly. A variable Hounsfield density of 230–260 HU was recorded; readings were more accurate where the carapace was thicker, especially inferiorly. The exterior appearance of the substance is rippled as though it has been placed wet over the textile. The substance has also been used to fill the space on both anterior and posterior aspects of the body, as if to pack it out.

**Table 1:** Carapace dimensions.

Measurement location	Thickness
Thickness at anterior maxilla midline	1.5mm
Thickness at anterior inferior thorax	7.0mm
Thickness at anterior mid-thigh	6.3mm
Posterior mid-thigh	25.0mm
Anterior ankles	3.3mm

#### μ-XRF and Raman analysis methods

To materially characterise the carapace, fragment NMR.27.13b was subjected to micro X-ray fluorescence (μ-XRF) and Raman analysis. The μ-XRF analysis was performed using a Bruker M4 Tornado, equipped with a rhodium X-ray source and two energy dispersive spectrometers (EDS). The analysis used a rhodium source set at 50 kV, 200 uA under vacuum (0mbar). The analytical spot size was 20 μm with a step size of 15 μm and each analysis point was acquired for 20 ms per step.

Raman spectra were taken from a series of spots on the surface and cross-section, to analyse and identify the mineralogy of the pigments and layers on the carapace samples. Analysis was performed on a Horiba Jobin Yvon LabRAM HR confocal Raman microscope, equipped with a Peltier-cooled CCD, and a Leica x50 objective. A 532 nm laser with a 600 g/mm grating was used. Each analysis acquisition time was 30 seconds and were repeated 10 times per spot.

#### μ-XRF results

**Fig 6:**
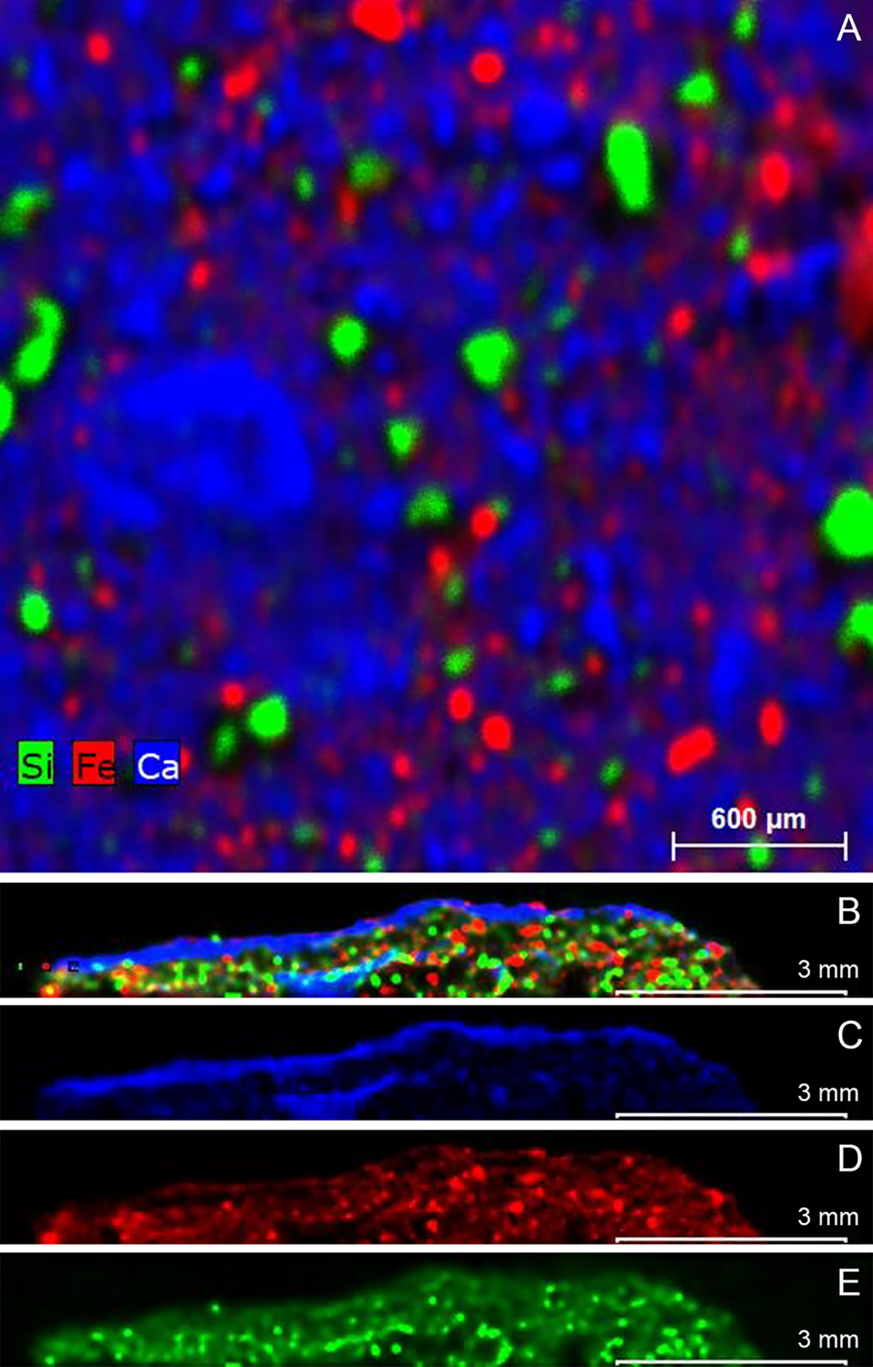
Carapace μ-XRF results (images by Timothy Murphy). A. Combined red, green and blue map of the red surface of the carapace, showing that the iron, calcium and silicon phases are separate. B–E. X-ray maps of carapace cross-section showing the distribution of iron (red) calcium (blue) and sulfur (green); scale bar: 3000 μm.

The surface of the carapace sample, coated in red pigment, was scanned and mapped using X-rays. The results reveal that the surface ochre layer comprised a mixture of Fe, Ca and Si (Fig 6A). There are also traces of minor elements including Mg, Al, P, S, K, Ti, Mn and Sr. Due to the interaction of the X-rays with surface material, it is possible the equipment also analysed sub-surface material. Therefore, the cross-section of the carapace sample was also scanned, as there are optically three thin yet distinct layers: red, white and brown material (Figs 2 and 3). The results (Fig 6B–6E) show that the red layer is made of Fe and Ca. The white layer is made of Ca, and the brown layer is a mixture of elements including Mg, Al, P, S, K, Ti, Mn and Sr. The white layer was originally hypothesised to be gypsum; however, no S was detected.

#### Raman results

Preliminary analysis of the surface layer showed that the red pigment consists of a mixture of minerals including haematite (Fe_2_O_3_), quartz (SiO_2_), ivory black (C) and some calcite (CaCO_3_). The white layer mostly comprises calcite (CaCO_3_) and some minor traces of quartz (SiO_2_). The brown layer has high fluorescence. As this is the ‘mud’ layer, it is assumed a number of alumina silicates and clays make up the material, which produced noisy spectra. This result suggests that the white calcium layer from the μ-XRF result was most likely created from a source of limestone.

### Radiocarbon dating

#### Radiocarbon dating methods

Two textile samples from the wrappings were subjected to AMS radiocarbon dating at the Centre for Accelerator Science, ANSTO at Lucas Heights, Sydney. Sample 1 (ANSTO Code OZK368) comprised 256 mg of woven linen textile taken from a small existing incision in the superficial wrappings over the anterolateral aspect of the left orbital region. The fragment came from a layer that was closest to the body at the inferolateral aspect of the left orbital cavity. Sample 2 (ANSTO Code OZK369), weighing 351 mg, was obtained through an existing incision in the superficial wrappings on the left anterolateral aspect of the neck. It was not possible to extract more preferable human tissue samples [13] owing to the complete state of preservation. Sterilised tweezers were used to extract small fragments of linen wrappings from under the outer layers.

A clean segment of each linen sample was sub-sampled for dating, then treated to remove contamination. While the samples were deliberately taken from inside the wrappings and therefore protected from external contaminants, possible contaminants may include embalming substances such as resins, oils, waxes, spices, wine and ancient salts. Therefore, the samples were pre-treated using a series of solvent extraction followed by bleaching with sodium chlorite in hydrochloric acid (2M) for two hours [8].

After chemical pre-treatment, samples were processed to graphite by combustion to CO_2_ at 900°C in sealed Vycor^TM^ tubes containing CuO and Ag wire. The CO_2_ was converted to graphite by reduction over Fe catalyst at 600°C using an excess of H_2_ [14]. The resulting graphite target was pressed into aluminium cathodes and analysed at ANSTO. The δ^13^C of the graphite was determined using an EA–IRMS [15]. The radiocarbon ages have been rounded according to Stuiver and Polach [16]. The unrounded radiocarbon ages were calibrated using OxCal 4.3.2 [17] and the IntCal 13 dataset of Reimer et al. [18].

#### Radiocarbon dating results

**Fig 7:**
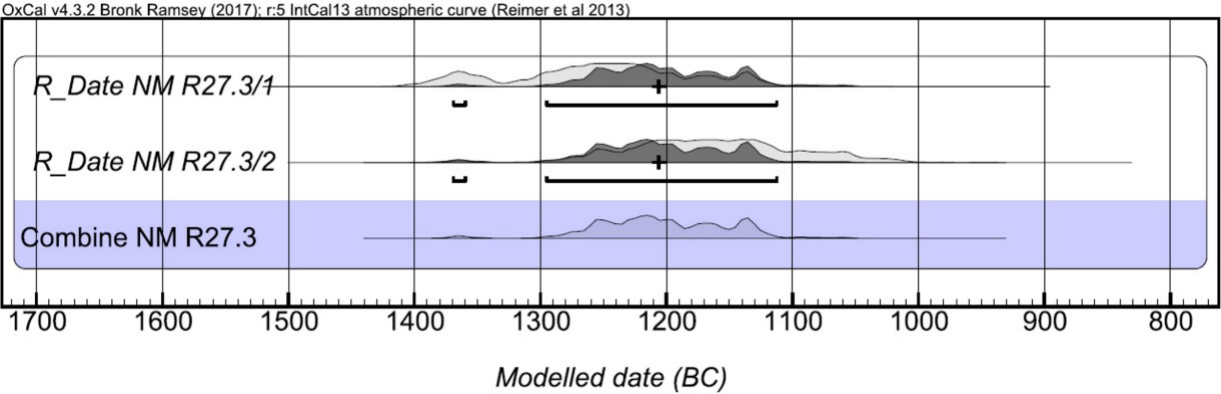
Probability distributions for calibrated ages for Sample 1, Sample 2 and for the combined results.

The results for samples OZK368 and OZK369 show close agreement (Table 2). As both samples were obtained from the same source, the two results were combined, giving a median date of 1207 cal BC and a range of 1370–1113 cal BC (95.4% probability). The probability distributions for Samples 1 and 2 and the combination of Samples 1 and 2 are shown in Fig 7.

**Table 2:** ^14^C dates from the linen wrappings of the mummified individual (NMR.27.3).

ANSTO code	Sample type	Museum no.	δ^13^C (‰)	^14^C content ±1 σ (pMC)	^14^C age ± 1 σ (BP)	Calibrated age range (2 σ, cal BC)
OZK368	Linen wrapping	NMR.27.3 Sample 1	-23.9 ± 0.1	68.76 ± 0.38	3010 ± 45	1396–1117
OZK369	Linen wrapping	NMR.27.3 Sample 2	-23.4 ± 0.1	69.21 ± 0.37	2955 ± 45	1286–1018

## Discussion

The above observations reveal that this individual, likely a female aged 26–35, was subjected to post-mortem treatments at various intervals across antiquity and modern times. The individual died, was mummified and wrapped in textiles in the late New Kingdom. The body was subjected to subsequent post-mortem damage in unknown circumstances, including the disarticulations described above. In an apparent attempt to repair and reunify the damaged body in antiquity, the individual was then subject to some rewrapping, packing and padding with textiles, and application of the mud carapace. Disarticulations, damage and repairs such as those described for the left knee and lower leg are determined to pre-date the carapace because they are deep to the shell. An exception here may be the disarticulation observed for the feet; although there are areas of discontinuity in the anterior and distal carapace, the textile wrapping is continuous. It therefore appears that the pedal disarticulation happened after the surrounding soft tissue desiccated and disintegrated. Around the head area at least, the mud surface was then painted with a base coat of white, possibly limestone-based pigment. The surface over the face was then coated with red-brown ground mineral pigment, as indicated by the mud carapace samples. At a later date, the body was damaged again in unknown circumstances on the right side and in the neck, cranial and facial regions. This phase of intervention is argued to be the most recent as the damage impinges on all underlying layers, including the carapace. Modern metal pins are observed to have been inserted in various places to stabilise the most recent damage. Commencing around the region of the greater trochanters, the pins are bilaterally distributed up towards the shoulders with a greater focus on the left side. They then extend mainly on the lateral aspects of the body and move superiorly to the left side of the head.

The wrappings date the mummification to c. 1370–1113 cal. BC. Although a wide dating span, the latter part of this date range aligns with the presence of dense packing in the thorax, a technique that emerged in the 13th century (19th Dynasty) and became increasingly common in the 12th–early 11th centuries (20th–early 21st Dynasties) [1 (p 90), 19 (p 64)]. This transition is seen in the mummification technique used for kings of the period. Owing to the later restoration and reburial of the royal mummies in ancient times, comparisons should be approached cautiously, but certain general trends are observable. The technique of placing linen packages, loose linen, resin and resin-impregnated linen in the torso is evident in the 18th Dynasty and also the 19th Dynasty [20 (p 227)]. The torsos of kings Seti I (date of reign c. 1294–1279 BC; Cairo Catalogue Général [CG] 61077) and Rameses II (c. 1279–1213 BC; CG61078) were both packed with resin-soaked bandages [1 (p 53), 20 (p 157–158)], while the torso of Merenptah (c. 1213–1203 BC; CG61079) was packed with ‘a white cheesy material’ (possibly a mixture of ‘fat and soda’, similar to that seen in 21st Dynasty mummification) [1 (p 67), 20 (169)], and the torso of Siptah (c. 1194–1188 BC; CG61080) was packed with lichen [1 (p 73)]. Packing with materials like lichen, mud, sand, dry earth, sawdust and/or linen pieces continued into the 21st Dynasty, as demonstrated by the mummified body of Horemkenesi [19 (p 64)].

The internal carapace on the mummified individual in Sydney likely fulfilled a threefold purpose. Firstly, it was a form of conservation for a body that had suffered significant post-mortem damage. The time between the mummification of the body, the burial, the first incident of damage, and the application of the carapace is likely to have been brief. Tombs and even simple graves were often robbed within a short time after interment of the deceased, as, despite the significance placed on afterlife beliefs throughout ancient Egyptian history, the obligation of the living to attend to the needs of the dead was generally short-lived in the non-royal context [21]. Those that were responsible for restoring the physical appearance of the mummified body after the first incident of damage were likely no more than one or two generations younger, and it can be assumed that the ^14^C dating of the linen fragments is also an appropriate range for the carapace. Evidence of ancient conservation on New Kingdom mummified bodies using a variety of means is well-known: the damaged body of king Seti I was re-wrapped more than once [20 (p 158–160, 220)], and king Amenhotep III (c. 1390–1352 BC; GC61074) may have been anciently restored using bandages, linen padding and re-packing the torso [1 (p 48, 49)]. Such techniques served to consolidate and restore bodies that had either deteriorated prematurely during the initial embalming process or had been later tampered with by tomb robbers [1 (p 48), 5 (p 122)]. Moreover, in the case of Amenhotep III and other bodies of kings and royal family members from the New Kingdom, mud was likely used as subcutaneous packing during the early stages of mummification to make the body more life-like. The packing, while not scientifically tested from these individuals, was probably a mixture of ingredients which likely included not only resin and mud, but also linen, natron, sawdust, soil, soda, sand and clay [22 (p 198–199].

Secondly, the carapace aided the metaphysical transition of the deceased into the afterlife and the sphere of the god Osiris. The mythological experiences of this underworld deity–his death, dismemberment, re-establishment and re-birth–had long been established to serve as a precedent for the mortuary experience of all Egyptians [23 (p 129), 24 (p 5–7)]. Like the god, the deceased could also hope for continued existence in the afterlife, when properly prepared. Part of that preparation involved the corporeal aspect of the deceased: analogous to Osiris, whose body had been torn apart and pieced back together, the death of the individual represented the disconnection of their various bodily components that then had to be restored through the act of mummification [25 (p 23–38)]. The embalming, wrapping and dressing of the body thus transformed the deceased into a being capable of joining Osiris in the hereafter and partaking in his immortality [24 (p 262, 377), 26]. In the case of the individual under study, the integrity of the mummified body had been compromised through post-mortem damage of unknown origin. The subsequent application of the mud carapace, in conjunction with some rewrapping and repacking, would have served to reunify the corporeal integrity of the deceased and ensure their continued association with Osiris. Mud may have been considered particularly effective in facilitating this process, given associations found in the textual and archaeological record between the Osiris’ re-birth and the renewed fecundity of Egypt’s agricultural soil following the Nile inundation [27 (p 223–225)].

Thirdly, the need for a restorative carapace on the mummified body may also have provided the opportunity to emulate elite funerary practices, as a form of status display by those responsible for the repair. Resin carapaces on the bodies of elite individuals are attested in the 19th and 20th Dynasties, and even in the 18th Dynasty, the skin of Amenhotep III may have been painted with a layer of resin as suggested by CT scans [20 (p 76)]. Seti I was described in the following terms: the ‘whole surface of the body, except the head only, is covered with a mass of bandage impregnated with resinous material’ [1 (p 57, plate xxxviii)]. Rameses II was similarly treated, including over the legs where the carapace was up to 6mm thick [1 (plate xlii)]. Merenptah was ‘covered in parts by a thin layer of very fine linen impregnated with a bright yellow resin-like material…the chest wall, parts of the leg and feet were enclosed in this balsam-impregnated carapace of fine linen’; in addition, ‘…a thin layer of red paste had been applied to the face’ [1 (p 66–67, plates xlvi–xlix)]. The bodies of kings Seti II (c. 1200–1194; CG61081) and Rameses III (c. 1184–1153 BC; CG61083) were likewise treated, with the ‘resin-impregnated carapace’ evident in an original photograph of the latter [1 (plate l)]. Not only did king Rameses V (c. 1147–1143 BC; CG 61085) feature viscera packed in sawdust inside the torso, but the face was also ‘painted an earthy red colour, like that of the mummies of many priests’ [1 (p 90)].

Shells have been reported on mummified bodies of lesser status but there is ambiguity in the data. An adult male apparently from the Theban area housed in the Redpath Museum, McGill University (RM2718), was reported with ‘three successive levels of linen bandages, with a layer of resin or plaster between them, from the shoulders to the mid-calf’, but the resin/plaster layer is hard to discern from the published images [28 (p 1239)]. The individual may have lived in the New Kingdom, but requires archaeometric dating [28 (p 1246)]. A study from the Turin Museum mentions ‘stucco plaster soaked linen wrappings’ but no further information is given [29 (p 598)]. Published transverse images of Padiamun (National Museums of Liverpool No. 53.72a) and Perenbast (Manchester Museum MM5053.a), both dating to the 22nd Dynasty (945–715 BC), reveal a dark grey layer within the wrappings which could be a mud carapace but this is not discussed [30 (p 97, figure 5.32; p 145, figures 5.196–199)].

While carapaces of resin were part of elite funerary practice as demonstrated by the mummified bodies of kings from this period, much remains to be understood about the precise nature of resin preparations used on these individuals. Scientific studies on other mummified bodies of different periods reveals that a variety of organic compounds were used [31]. These included ingredients imported from the eastern Mediterranean such as conifer and pistacia (ancient Egyptian *sn**t**r*) resins, used extensively in New Kingdom elite embalming [22, 32]. People of lesser means had more limited recourse to expensive imported resins, especially in the quantities needed to create a protective shell over the body. However, emulation of elite burial practices could be attained through the use of cheaper, locally available alternatives. In the case of the individual under study, the use of mud for the carapace may not only have had perceived regenerative properties but would have been an expedient and low-cost solution.

Like the carapace itself, the presence of red colouring over the face recalls the 19th–20th Dynasty mummification techniques of Merenptah and Rameses V, noted above. The faces of later mummified male individuals from the 21st Dynasty were commonly painted red while those of females were painted yellow [5 (p 127)], reflecting the traditional gendered skin colouring used in Egyptian art. The presence of red pigment, rather than yellow, on the face of the carapace would not necessarily undermine the ‘probable female’ assignation established through this investigation. Representations of females with dark red-brown skin colouring are known from the Theban funerary context of the 19th and 20th Dynasties, including a group of non-royal coffins and mummy boards from Deir el-Medina (e.g. Brooklyn Museum 37.47Ea–d, Cairo Museum JE27309, Metropolitan Museum of Art 86.1.5c, National Museums Scotland A.1887.597) and depictions of royal women in their tombs in the Valley of the Queens [33 (p 191–193)].

## Conclusion

Based on radiocarbon dates, the mummified individual NMR.27.3 originated from the late New Kingdom, with a range of c. 1370–1113 cal BC and a median of 1207 cal BC. The technique of packing the torso appears toward the end of the 13th century BC, suggesting the individual should be placed in the 12th century BC rather than the earlier end of the radiocarbon range.

CT scans and extracted samples revealed the use of a mud carapace around the mummified body. The face was painted red and the whole carapace may have been thus decorated. While resinous shells are recorded on late New Kingdom royal mummified remains, casings made of mud are not thus far scientifically documented and dated. Thus, the mummified individual under study may represent a unique window into the phenomenon of elite emulation in the burial customs of non-royal Egyptians. The multi-proxy nature of this study will assist in the identification of other carapaces, a feature likely more common in Egyptian mummification than hitherto recorded.

Moreover, the carapace also served as an ancient form of restoration for a damaged body, offering further insights into not only the extent of technical corporeal intervention and posthumous maintenance of the dead, but also the seriousness with which Osirian afterlife beliefs concerning the integrity of the body were held. Although the social position of the individual cannot be determined, those caring for this person in death were of sufficient means to afford complex embalming including evisceration, internal packing and linen wrappings. They, or others associated with the deceased, had enough concern for the latter’s posthumous well-being to later invest in a mud-plastered and painted carapace after the body had been disturbed and dismembered. Understanding how common this practice had become in the late New Kingdom will require the radiological study and publication of further non-royal mummified individuals from this era.
